# Tert-Butylhydroquinone Mitigates T-2-Toxin-Induced Testicular Dysfunction by Targeting Oxidative Stress, Inflammation, and Apoptosis in Rats

**DOI:** 10.3390/toxics12050335

**Published:** 2024-05-05

**Authors:** Yun Chen, Xinke Zhang, Shanshan Lan, Shuping Liang, Manyu Zhang, Shuang Zhang, Yijian Liu, Li Li, Hengxi Wei, Shouquan Zhang

**Affiliations:** 1 Guangdong Provincial Key Laboratory of Utilization and Conservation of Food and Medicinal Resources in Northern Region, College of Biology and Agriculture, Shaoguan University, Shaoguan 512005, China; luxixiworld@163.com (Y.C.); lisablue0729@163.com (S.L.); l18320286776@163.com (S.L.); 15815800972@163.com (M.Z.); 18573430419@163.com (S.Z.); 13129669109@163.com (Y.L.); 2 Guangdong Provincial Key Laboratory of Agro-Animal Genomics and Molecular Breeding, College of Animal Science, South China Agricultural University, Guangzhou 510642, China; 15036784245@163.com (X.Z.); lili007007@scau.edu.cn (L.L.)

**Keywords:** T-2, tert-butylhydroquinone, testis, oxidative stress, apoptosis, inflammation

## Abstract

Tert-butylhydroquinone (tBHQ) has emerged as a promising candidate for mitigating the adverse effects of T-2-induced reproductive toxicity. The protective effects of tBHQ on rat sperm quality, testicular injury, apoptosis, and inflammation induced by T-2 toxin exposure were investigated. Histopathological examination of testicular tissues revealed severe damage in the T-2-treated group, characterized by disorganized germ cell arrangement, thinning of the convoluted seminiferous tubule walls, and significant cellular necrosis. However, tBHQ administration, either as a preventive or therapeutic measure, mitigated this structural damage. Image analysis confirmed an increase in the cross-sectional area and height of the convoluted seminiferous tubules in the tBHQ-treated groups compared to the T-2-treated group (*p* < 0.05), indicating tBHQ’s efficacy in alleviating testicular damage. Additionally, tBHQ treatment significantly inhibited T-2-induced apoptosis of testicular tissue cells, as evidenced by the results showing reduced apoptotic cell counts and downregulation of the BAX/BCL2 ratio and caspase-3 expression (*p* < 0.05). tBHQ significantly increased the concentrations of the antioxidant factors SOD, CAT, TAC, and GSH-PX. Furthermore, tBHQ attenuated the inflammatory response induced by T-2 exposure, as indicated by the decreased mRNA expression of the proinflammatory cytokines *Tnf*, *Il1*, and *Il10* in testicular tissue (*p* < 0.05). Additionally, tBHQ treatment alleviated the decline in serum testosterone induced by the T-2 and promoted testosterone synthesis gene expression, including for the genes *17β-HSD* and *Cyp11a1*, in rat testes (*p* < 0.05). These findings underscore tBHQ’s role as a therapeutic agent combatting T-2-induced reproductive toxicity, highlighting its antioxidative, anti-apoptotic, and anti-inflammatory properties. Further elucidation of tBHQ’s mechanisms of action may offer novel strategies for preventing and treating reproductive disorders induced by environmental toxins.

## 1. Introduction

In the fields of environmental pollution and food safety, fungal toxins are recognized as a significant health hazard. Among these toxins, T-2 toxin (T-2) is a prevalent toxic compound found abundantly in the natural environment, particularly in grains and animal feed [1]. Produced by *Fusarium* molds, this toxin represents a potent biologically active substance posing a severe risk to health. T-2 can induce immunosuppression, cytotoxicity, and reproductive toxicity. Recent research has highlighted its capacity to induce reproductive tract damage and impair reproductive capacity [2]. However, the precise mechanisms underlying its toxicity remain unclear. Studies suggest that T-2-induced testicular damage may be mediated by the induction of oxidative stress [3,4]. Its destructive toxic effects primarily involve cellular damage to testicular tissue, disruption of spermatogenesis, and an eventual decline in reproductive capability [5]. Reactive oxygen species (ROS) can directly or indirectly impact intracellular apoptotic signaling pathways, activating pathways involving apoptotic enzymes and ultimately prompting cellular apoptosis [6]. In testicular tissue, oxidative stress directly harms critical cell types involved in sperm formation, such as spermatogenic and interstitial cells, thereby ultimately affecting the quantity and quality of sperm [7].

In response to the damage induced by oxidative stress, biological processes increase the activity of endogenous antioxidant defense systems, including various antioxidant enzymes and small-molecule antioxidants [8]. These antioxidant substances can neutralize free radicals and stabilize the redox balance within cells [9]. However, increased exposure to environmental toxins, such as T-2, may overwhelm these endogenous antioxidant systems, exacerbating oxidative damage within testicular tissue [10]. Tert-butylhydroquinone (tBHQ), as a potential antioxidant, has garnered significant attention from scientists [11]. tBHQ has the ability to scavenge free radicals and boost the activity of antioxidant enzymes within cells, thereby effectively mitigating cellular damage caused by oxidative stress [12]. It holds promise in the prevention and treatment of T-2-induced cellular injuries. Due to its low cost and potent antioxidant activity achievable at relatively low doses, tBHQ has garnered significant interest in recent years. Research findings have elucidated its protective role in testicular injury [13,14].

Therefore, this study aims to investigate whether tBHQ can alleviate T-2-induced testicular injury and low sperm quality by reducing oxidative damage and apoptosis pathways. We hypothesize that tBHQ may inhibit the production of ROS and activation of the caspase family proteins, thereby preventing T-2-induced oxidative stress and cell apoptosis, ultimately safeguarding the testes and sperm quality of rats.

## 2. Materials and Methods

### 2.1. Reagents

T-2 was purchased from J&K Scientific Technology Co., Ltd. (CAS:21259201, 902030, 98% pure, Beijing, China). tBHQ was purchased from J&K Scientific Co., Ltd. (CAS:1948330, B0833, 98% GC, Beijing, China). Trizol was purchased from Invitrogen (Carlsbad, CA, USA), while the prescript RT reagent kit and SYBR Green I fluorescent dyes were purchased from Invitrogen. A BCA protein assay kit and assay kits for measuring total antioxidant capacity (T-AOC) and superoxide dismutase (SOD), glutathione peroxidase (GSH), catalase (CAT), and malondialdehyde (MDA) levels were purchased from Nanjing Jiancheng Biotech (Nanjing, China). The primary antibodies for Bax (Catalog number: PAB343Ra01), Bcl-2 (Catalog number: PAA778Ra01), and caspase-3 (Catalog number: PAA626Ra01) were obtained from Cloud-Clone Corp (Wuhan, China). The primary antibodies for GAPDH (T0004), cleaved-caspase-3 (Asp175), STAT3 (Catalog number: AF6294), JAK2 (Catalog number: AF6022), p-STAT3 (Catalog number: AF3293), p-JAK2 (Catalog number: AF3022), and p-caspase-3 (AF3311) were obtained from affinity Biosciences (SLC, UTAH, USA). CV value, also known as the coefficient of variation, is expressed as a percentage, and it is the ratio of the standard deviation to the mean. FSH: Catalog number: RX302805R, RUIXIN BIOTECH. Detection range: 1.25→40 mIU/mL. CV values for intra-assay variability are all <10%, and inter-assay variability values are all <15%. LH: Catalog number: RX302644R, RUIXIN BIOTECH. Detection range: 1.5→48 mIU/mL. CV values for intra-assay variability are all <10%, and inter-assay variability values are all <15%. GnRH: Catalog number: RXJ302798R, RUIXIN BIOTECH. Detection range: 5–80 mIU/mL. CV values for intra-assay variability are all <15%, and inter-assay variability values are all <15%.

Testosterone: Catalog number: HEA458Ge, Cloud-Clone Corp. Detection range: 2.47–200 pg/mL. Intra-assay variability: CV < 10%; inter-assay variability: CV < 12%.

TNF-α (Rat): Catalog number: ER1393, FineTest. Detection range: 3.906–250 pg/mL. Intra-assay variability: CV < 8%; inter-assay variability: CV < 10%. Catalase (CAT): Catalog number: A007-1-1, Method: Ammonium molybdate method, and Company: Nanjing China Jiancheng Bioengineering Institute. Detection range: 0.2–24.8 U/mL. Intra-assay variability: CV < 1.9%; inter-assay variability: CV < 4.94%. Total antioxidant capacity (T-AOC): Catalog number: A015-2-1, Method: ABTS method, Company: Nanjing Jiancheng Bioengineering Institute (Nanjing, China). Detection range: 0–1 mM. Intra-assay variability: CV < 5%, inter-assay variability: CV < 10%. Superoxide dismutase (SOD): Catalog number: A001-3-2, Method: WST-1 method, Company: Nanjing Jiancheng Bioengineering Institute. Detection range: 0.5–122.1 U/mL. Intra-assay variability: CV < 5.50%, inter-assay variability: CV < 3.32%. Glutathione peroxidase (GSH-PX): Catalog number: A005-1-2, Method: Colorimetric method, Company: Nanjing Jiancheng Bioengineering Institute. Detection range: 20-330U. Intra-assay variability: CV < 3.1%, inter-assay variability: CV < 4.34%. Malondialdehyde (MDA): Catalog number: A003-1-1, Method: TBA method, Company: Nanjing Jiancheng Bioengineering Institute. Detection range: 0–113.0 nmol/mL. Intra-assay variability: CV < 3.5%, inter-assay variability: CV < 4.11%.

Enhanced chemiluminescence (ECL) reagent was purchased from Nanjing KeyGen Biotech. Co., Ltd. (Nanjing, China). A rat testosterone ELISA kit was purchased from Wuhan Cloud Clone Co., Ltd. (Wuhan, China).

### 2.2. Animals

Specific pathogen-free male Sprague-Dawley rats (6 weeks old, 150 ± 10 g) were purchased from the Guangdong Experimental Animal Center in Guangzhou, Guangdong Province, China. All rats were housed in animal facilities with a room temperature of 18–22 °C, relative humidity of 40–60%, and a 12 h light/dark cycle. Food and water were provided ad libitum for all rats. Prior to the commencement of the experiment, all animals were acclimatized to the animal housing environment for one week. The investigation into the preventive and therapeutic effects of tBHQ on mitigating T2 reproductive damage, aimed at determining its efficacy and safety, was conducted using the following groupings. The animals were randomly divided into four groups: control group (n = 10, employing regular feeding, with diluted solution injected for 21 days), T-2 group (n = 10, 0.05 mg/kg, diluted solution injected for the first 14 days, followed by oral gavage of T-2 for the next 7 days), preventive group (tBHQ+T-2, n = 10, 50 mg/kg tBHQ injected intraperitoneally for the first 14 days, followed by oral gavage of T-2 for the next 7 days), and mitigation group (M group, n = 10, injected with diluted solution for the first 14 days, with oral gavage of T-2 and injection of tBHQ at the same time for the next 7 days; tBHQ dosage was 50 mg/kg/day in 1% DMSO-PBS solution, and T-2 dosage was 0.05 mg/kg/day in corn oil). No animals suffered unnecessary pain during the experiment. The experimental procedures were conducted following the China Council on Animal Care guidelines and approved by the Animal Care Committee of Shaoguan University (approval number: 2022-11sc-002). After an overnight fast of 15 h, all rats were administered pentobarbital sodium through intraperitoneal injection before blood collection from the eyeball, followed by rapid removal and collection of testicular and epididymal tissue samples. The testicular tissue was partitioned into four segments: one segment was immersed in a 10% formalin solution for subsequent histological examination, while the remaining segments were stored at −80 °C pending further analysis.

### 2.3. Oxidative Stress Indicators

Testis tissues were tested by using MDA, SOD, and CAT kits and GSH-Px kits, which were used according to the manufacturer’s instructions. Five testis tissue samples were tested for these indicators in each treatment group. The color development solution was prepared and incubated with the development solution, and the OD value of the samples were measured. MDA was detected at 532 nm, SOD was detected at 550 nm, CAT and GSH were detected at 405 nm, and GSH-PX was detected at 412 nm.

### 2.4. Sperm Motility

The rat epididymides were placed in a 24-well plate filled with preheated PBS. Once the sperm had completely exited the epididymides, 10 µL of the sperm suspension was extracted for analysis on a sperm-count plate. The count of viable sperm and the total count of sperm were recorded, and subsequently the sperm viability ratio was computed (viable sperm count/total sperm count × 100%).

### 2.5. Histopathology of Testicular Tissue

The testicular tissues underwent fixation in a 10% formalin solution for a duration of 2–3 days, followed by successive dehydration in varying concentrations of alcohol. Subsequently, they were embedded in paraffin, sectioned, and sliced into 5 µm thick sections. These slices were subjected to staining with hematoxylin and eosin (H&E) to facilitate histological examination. After staining, the sections were mounted and observed under a virtual optical microscope with a magnification factor of 200× (model BX51TF, Olympus Corporation, Tokyo, Japan), while images were analyzed using Image Pro-Plus Motic Med 6.0 software (Motic Incorporation, Xiamen, China).

### 2.6. TUNEL Staining

To evaluate apoptosis in rat testicular tissues, a TUNEL assay was employed. Testicular tissue sections, measuring 4 µm in thickness, were initially rinsed with PBS. Subsequently, they underwent permeabilization with proteinase K at 37 °C for 30 min, followed by triple PBS rinses at room temperature for 5 min each. The sections were then subjected to incubation with TdT and dUTP at 37 °C for 2 h. After another round of PBS rinses, the tissue sections were examined using fluorescence microscopy (Nikon, Eclipse C1, Tokyo, Japan). Image analysis was conducted on five randomly selected areas of each section, with a magnification of 200×. (Note: DAPI emits blue light with an excitation wavelength of 330–380 nm and an emission wavelength of 420 nm, while CY3 emits red light with an excitation wavelength of 510–561 nm and an emission wavelength of 590 nm.) The quantities of DAPI- and TUNEL-positive dots were calculated using image pro software (V2.2.5), presenting DAP-positive points as the total number of cells and TUNEL-positive points as the channel points. Finally, TUNEL/DAPI × 100% was used to calculate the ratio of apoptotic cells.

### 2.7. Western Blot Analysis

The testicular tissues were extracted and transferred into a centrifuge tube containing RIPA lysis buffer supplemented with phenylmethanesulfonyl fluoride (PMSF). Subsequently, the homogenate was subjected to centrifugation at 12,000× *g* for 5 min at 4 °C. Following the collection of the Western blot supernatant, a BCA protein assay kit was utilized to ascertain the total protein concentrations. Each sample, containing 20 mg of lysate, was loaded onto a 12% SDS-PAGE gel for electrophoresis and then transferred to PVDF membranes (Millipore, Bedford, MA, USA). For Western blotting, proteins were first separated via 10% sodium dodecyl sulfate polyacrylamide gel electrophoresis (SDS-PAGE) and subsequently transferred onto PVDF membranes (Millipore, Bedford, MA, USA). After blocking with a 5% nonfat milk–TBST buffer for 1 h at 4 °C, the membranes were incubated overnight at 4 °C with primary antibodies, including GAPDH, caspase-3, cleaved-caspase-3, Bax, Bcl-2, STAT3, p-STAT3, JAK2, and p-JAK2. Following incubation, the membranes were washed three times with TBST at room temperature and then incubated with the corresponding secondary antibody for 1 h. Finally, protein bands were visualized using an ECL reagent and quantitatively analyzed with Image-Pro Plus 6.0 software (BioRad, Hercules, CA, USA).

### 2.8. Q-PCR

Rat testicular RNA extraction was conducted, and the expression of hormone-synthesizing genes was assessed via Q-PCR. Total RNA extraction from tissue samples was carried out using TRIzol (Invitrogen, Carlsbad, CA, USA), following the manufacturer’s instructions. The concentration of RNA was determined using the Thermo ND2000 (Thermo, San Francisco, CA, USA), and reverse transcription to cDNA was performed using Superscript III (Invitrogen). Each reaction utilized 1 μg of RNA template for cDNA synthesis, following the manufacturer’s protocols. Amplification was executed using the CFX Opus 384 (BioRad, Alfred Nobel Drive, Hercules, CA, USA), comprising 40 cycles of denaturation at 95 °C for 10 s, annealing at 58 °C for 10 s, and extension at 72 °C for 30 s. Each Q-PCR analysis was conducted with quadruplicate biological replicates. The 2^−ΔΔCT^ method was employed to assess the relative mRNA expression levels, with GAPDH serving as the reference gene. Please refer to Table S1 in Supplementary Materials for the gene sequences.

### 2.9. Statistical Analysis

The experimental data for each group were analyzed using SPSS 21.0 statistical software (Release 21.0; SPSS, Inc., Chicago, IL, USA), using one-way ANOVA with Tukey’s test. Statistical analysis was performed using GraphPad Prism 7.0 software (San Diego, CA, USA). The results are expressed as means ± SEM. *p* values < 0.05 were considered statistically significant, while *p* values < 0.01 were considered highly significant.

## 3. Results

### 3.1. tBHQ Alleviates T-2-Induced Decline in Sperm Quality and Testicular Injury

**Figure 1:**
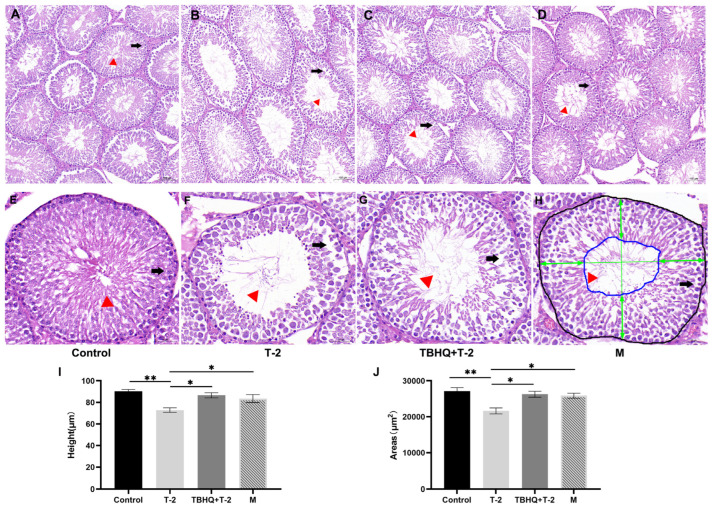
tBHQ alleviates T-2 induced testicular injury. (**A**–**D**) are at 200× magnification, with a scale bar of 100 μm, while (**E**–**H**) are at 400× magnification, with a scale bar of 50 μm. (**A**,**E**) represent the control group, (**B**,**F**) represent the T-2 group, (**C**,**G**) represent the tBHQ+T-2 group, and (**D**,**H**) represent the M group. Testicular tissue sections stained with hematoxylin and eosin (HE) were observed under a light microscope. In the control group, germ cells were orderly arranged in various layers, with thick walls of the convoluted seminiferous tubules and abundant spermatozoa in the tubular lumens at the locations indicated by the red-boxed triangles in (**A**,**E**). Conversely, in the experimental groups, germ cells were loosely arranged and disorganized, with thinning walls of the convoluted seminiferous tubules and reduced numbers of mature spermatozoa in the tubular lumens at the locations indicated by the red-boxed triangles in (**B**–**D**,**F**,**H**). Under high magnification, observations of the T2 group revealed basal membrane disruptions in the walls of the convoluted seminiferous tubules (indicated by the black arrow in (**B**)), with numerous germ cells being detached from the tubular lumens (indicated by the black arrow in (**F**)), and the black-lined area in (**H**) defines the cross-sectional area of the convoluted seminiferous tubules. The area within the blue lines defines the luminal area. The average length of the four green double-headed arrows represents the height of the germinal epithelium. (**I**) Cross-sectional area of the convoluted seminiferous tubules. (**J**) Height of the convoluted seminiferous tubules. n = 8. * represents a significance difference, with *p* < 0.05. ** represents a significance difference (*p* < 0.01).

T-2 significantly decreased the sperm motility of rats compared to the control, and the rats in tBHQ treatment and prevention groups exhibited significantly increased sperm motility after being challenged with T-2 (Table 1). Histopathological examination of testicular tissues under HE staining revealed distinct differences between the control and experimental groups. In the control group, various stages of germ cells were orderly arranged, and the walls of convoluted seminiferous tubules appeared thick, harboring a considerable number of spermatozoa within the tubular lumens (Figure 1A,E, indicated by the red-boxed triangle). Contrastingly, in the experimental group, the arrangement of germ cells in testicular tissues was loosely disorganized, and the walls of convoluted seminiferous tubules exhibited thinning, accompanied by a reduced quantity of mature spermatozoa within the tubular lumens (Figure 1B–F,H, indicated by the red-boxed triangle). Under high magnification, observation of the T2 group revealed disruptions in the basal membrane of the walls of the convoluted seminiferous tubules (Figure 1B, indicated by the black arrow), with a significant detachment of numerous germ cells from the tubular lumens (Figure 1F, indicated by the black arrow). The results indicate that the T-2-treated group exhibited damage to the structure of convoluted seminiferous tubules. Furthermore, the tBHQ+T-2 and M group demonstrated a reduction in structural damage to the convoluted seminiferous tubules.

The results revealed a significant decrease in the cross-sectional area and height of the convoluted seminiferous tubules in the T-2-treated group compared to the control group. Furthermore, compared to the T-2 group, the tBHQ+T-2 and M groups exhibited a marked increase in the cross-sectional area and height of the convoluted seminiferous tubules.

### 3.2. tBHQ Alleviates T-2-Induced Oxidative Stress

After administering the T-2 treatment to the rats, the concentration of MDA in their testicular tissue significantly increased. However, both the treatment and preventive groups treated with tBHQ exhibited a significant decrease in MDA concentration (Table 1, *p* < 0.05). Additionally, compared to the T-2 group, the treatment group showed a significant increase in the concentrations of the antioxidant factors SOD, CAT, TAC, and GSH-PX (Table 1, *p* < 0.05).

### 3.3. tBHQ Inhibits T-2-Induced Apoptosis of Testicular Cells

**Figure 2:**
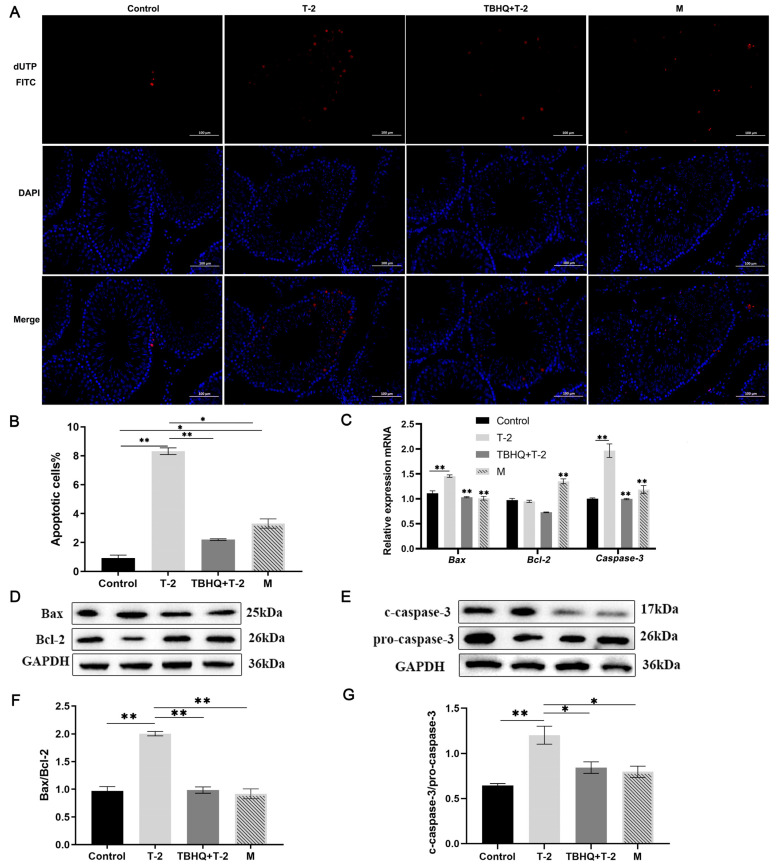
tBHQ inhibits T-2-induced apoptosis of testicular cells. (**A**) TUNEL staining; (**B**) semi-quantitative analysis of apoptotic cell ratio using TUNEL staining fluorescence; (**C**) mRNA expression levels of apoptotic factors Bax, Bcl2, and Caspase-3; (**D**–**F**) protein expression levels of Bax and Bcl2, represented by grayscale values; (**E**–**G**) protein expression levels of Caspase-3, represented by grayscale values. n = 10. * represents a significant difference, with *p* < 0.05. ** represents a significant difference, with *p* < 0.01.

It is evident that T-2 treatment significantly augments the apoptosis of testicular tissue cells at the cellular level. Notably, administration of tBHQ, either as a preventive measure or therapeutic intervention, markedly mitigates testicular cell apoptosis (Figure 2A,B, *p* < 0.05). At the molecular level, T-2 induces a substantial increase in the ratio of BAX to BCL2, along with elevated mRNA and protein expression levels of caspase-3 (Figure 2C–G, *p* < 0.05).

### 3.4. tBHQ Alleviates the Inflammatory Response Induced by T-2

**Figure 3:**
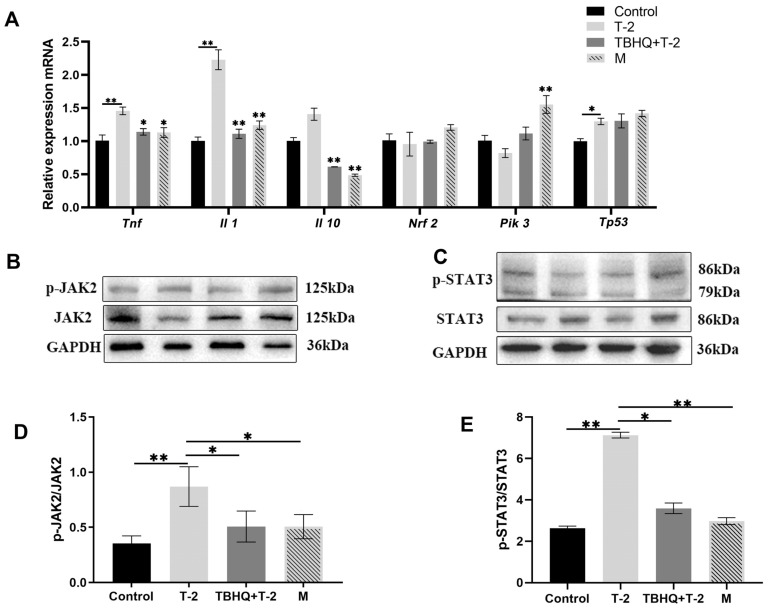
tBHQ alleviates the inflammatory response induced by T-2. (**A**) mRNA expression levels of inflammatory factors; (**B**,**D**) protein expression levels and phosphorylation of JAK, represented by grayscale values; (**C**,**E**) protein expression levels and phosphorylation of STAT3, represented by grayscale values. n = 8. * represents a significant difference, with *p* < 0.05. ** represents a significant difference, with *p* < 0.01.

Analysis of inflammation factor expression in testicular tissue and serum revealed that T-2 significantly upregulates mRNA expression of the proinflammatory cytokines Tnf, Il1, and Tp53 within the testes while showing no significant difference in terms of the mRNA expression of Nrf2 (Figure 3A). At the protein level, there was a notable enhancement in the phosphorylation of JAK2 and STAT3 (Figure 3B–E, *p* < 0.05) induced by T-2 exposure.

### 3.5. tBHQ Attenuates T-2-Induced Decrease in Serum Testosterone Levels and Promotes Expression of Testosterone Synthesis Genes

**Figure 4:**
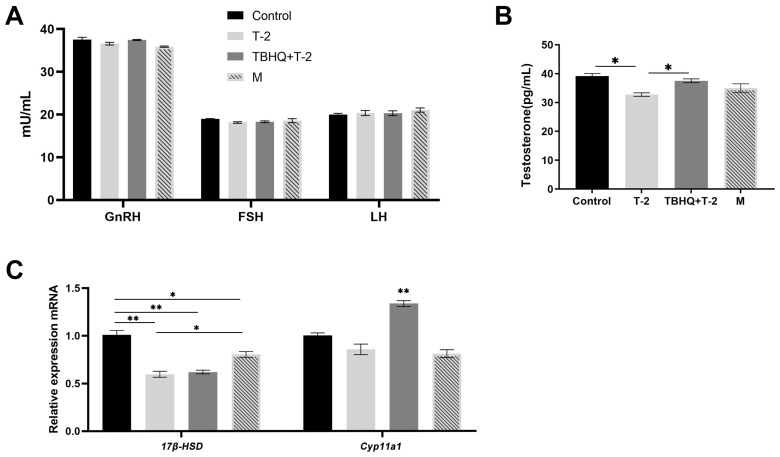
tBHQ Attenuates T-2-induced decrease in serum testosterone levels and promotes expression of testosterone synthesis genes. (**A**) Concentrations of Gnrh, FSH, and LH in rat serum. (**B**) Concentration of testosterone in serum. (**C**) mRNA expression levels of steroid hormone synthesis genes. n = 10. * represents a significant difference, with *p* < 0.05. ** represents a significant difference for *p* < 0.01.

T-2 had no significant impact on GnRH, FSH, and LH concentrations in rat serum (Figure 4A, *p* > 0.05). However, it significantly decreased the level of testosterone in rat serum (Figure 4B, *p* < 0.05). Administration of tBHQ, a preventive measure, alleviated the adverse effects of T-2. Analysis of testosterone synthesis gene expression in rat testes revealed a significant elevation in the mRNA levels of 17β-HSD in group M and Cyp11a1 in the tBHQ+T2 group upon administering the tBHQ treatment (Figure 4C).

## 4. Discussion

The fungal toxin T-2 is frequently encountered as an environmental contaminant and poses a significant threat to food safety. It is widely present in grains and feed, generating severe health impacts on humans and animals [1]. Studies have indicated that T-2 possesses immunosuppressive, cytotoxic, and reproductive-toxicity-inducing effects, with particular concern regarding its reproductive toxicity, although the underlying mechanisms remain unclear [2,15]. In this study, we observed the adverse effects of T-2 exposure on rat testicular tissue structure impairment and decreased sperm vitality. Further analysis revealed that T-2-induced oxidative stress and cell apoptosis, accompanied by inflammatory responses, led to cellular damage in rat testicular tissues. Oxidative stress directly damages vital cell types in sperm formation, thus affecting sperm quantity and quality. Despite the complex array of endogenous antioxidant defense systems in organisms, these defense mechanisms may be insufficient to counteract the oxidative pressure induced by T-2 [16]. In this study, we further investigated the mechanism of action of tBHQ as an antioxidant in T-2-induced testicular damage. The experimental results demonstrated that tBHQ effectively alleviated the damage to rat testicular tissue structure induced by T-2, reduced apoptosis and oxidative stress levels, and ameliorated the inhibition of the expression of the testosterone synthesis gene *17β-HSD* within the testes induced by T-2. This suggests that tBHQ effectively mitigated reproductive toxicity induced by T-2 by modulating the apoptotic pathway and protecting testicular tissue from oxidative stress damage.

At the tissue level, we observed significant testicular tissue damage and oxidative stress caused by T-2, accompanied by reduced serum testosterone levels in rats. Both the prevention group and the treatment group exhibited significant alleviations in the levels of oxidative stress caused by T-2. Testosterone, a key molecule in male sex hormones, plays a crucial role in sperm production and sexual function [17]. Therefore, the decrease in testosterone levels induced by the T-2 may significantly contribute to poor sperm quality. However, the administration of tBHQ in a preventive form significantly increased serum testosterone levels, indicating the notable protective effect of tBHQ.

Furthermore, we conducted an in-depth investigation into T-2-induced apoptosis.

Apoptosis is a vital mechanism of cellular demise that could potentially have a considerable impact on T-2-induced testicular injury [15]. At the cellular level, we observed a significant increase in apoptosis of testicular tissue cells following T-2 treatment. At the same time, both preventive and therapeutic administration of tBHQ significantly reduced cell apoptosis. The apoptotic factors Bax and Bcl-2 and their ratio play pivotal roles in regulating cell apoptosis. Bax acts as a pro-apoptotic factor, promoting cell apoptosis when overexpressed, whereas Bcl-2 is an anti-apoptotic factor, inhibiting cell apoptosis when overexpressed [18]. Hence, the Bax/Bcl-2 ratio balance is crucial in determining the equilibrium between cell survival and death [19]. Additionally, caspases are a critical class of apoptotic executioner proteins, playing vital roles in executing various stages of cell apoptosis, such as nuclear DNA degradation and cellular structure disintegration [20]. According to the research findings, the mRNA and protein abundances of Bax and Caspase were significantly increased in the T-2 treatment group, resulting in an increased Bax/Bcl-2 ratio, indicating an exacerbation of cell apoptosis induced by the T-2 treatment. In contrast, in the tBHQ herapeutic groups, Bax and caspase abundance was significantly decreased, while Bcl-2 levels were notably increased, leading to a decreased Bax/Bcl-2 ratio, suggesting that the application of tBHQ effectively suppressed T-2-induced cell apoptosis. This result suggests that tBHQ may protect cells from T-2-induced damage by modulating Bax, Bcl-2, and caspase expression levels. These results further validate the protective effect of tBHQ on testicular tissue.

*Tnf* and *Il-1* are classical proinflammatory cytokines that stimulate inflammation and play crucial roles in immune regulation. Mainly, they activate various cell types, including immune and endothelial cells, leading to the release of inflammatory mediators and the induction of biological effects such as vasodilation [21]. In contrast, *Il-10* serves as an anti-inflammatory cytokine, primarily inhibiting the production of proinflammatory cytokines and the activation of immune cells, thereby regulating the magnitude of the inflammatory response [22]. Consequently, the interaction between *Tnf*, *Il-1*, and *Il-10* maintains a balance in inflammation regulation, ensuring the stability of an organism’s internal environment. The JAK2/STAT3 signaling pathway is implicated in various physiological and pathological processes, including cancer, inflammation, and tissue injury. In inflammation, JAK2/STAT3 activation produces proinflammatory cytokines and mediates immune responses [23]. In this study, we observed variations in the mRNA levels of *Tnf*, *Il-1*, and *Il-10* and the phosphorylation levels of JAK2 and STAT3 proteins, with significant increases noted in the T-2 treatment group. In contrast, marked decreases were observed in the tBHQ preventive and therapeutic groups. These findings suggest that T-2 treatment may lead to the abnormal activation of inflammatory responses and cell signaling pathways. At the same time, tBHQ helps restore normal regulation of these pathways, thereby protecting an organism from damage.

At the molecular level, we further investigated the expression levels of critical genes associated with testosterone synthesis. The results revealed that in the tBHQ preventive or therapeutic groups, there was a significant increase in mRNA levels of *17β-HSD* and *Cyp11a1*. *17β-HSD* is an important enzyme that plays a crucial role in the androgen synthesis pathway, while *Cyp11a1* is one of the rate-limiting enzymes in testosterone synthesis. Therefore, by increasing the expression levels of these critical genes, tBHQ may promote testosterone synthesis, thereby alleviating testicular damage and the decrease in sperm quality induced by T-2.

While this study demonstrates the potential of tBHQ in mitigating the adverse effects of T-2-induced reproductive toxicity in rats, several limitations should be considered. Firstly, this study primarily focused on acute effects, and the long-term effects of tBHQ treatment on reproductive function remain unclear. Additionally, this study was conducted using a rat model, and extrapolating these findings to human populations requires further investigation. Furthermore, more in-depth exploration of the mechanisms underlying tBHQ’s protective effects is needed to fully understand its mode of action and potential side effects. Moreover, this study did not explore the potential interactions of tBHQ with other environmental toxins or medications, which could influence its efficacy and safety profile. Finally, while tBHQ showed promising results in regard to mitigating testicular damage, the determination of its translational potential as a therapeutic agent for human reproductive disorders requires rigorous clinical trials to establish safety and efficacy.

## 5. Conclusions

In summary, the results of this study indicate that tBHQ alleviates T-2-induced testicular damage and declines in sperm quality by modulating the expression levels of testosterone synthesis genes. Additionally, tBHQ protects testicular tissue from oxidative stress damage by inhibiting the activation of the apoptotic pathway. However, although we observed these significant protective effects, tBHQ’s exact mechanism of action still requires further investigation. Future research could delve into the interactions between tBHQ and other key signaling pathways, as well as its potential application in the clinical treatment of reproductive toxicity induced by T-2.

## Figures and Tables

**Table 1:** Testis relative weight, sperm motility, and antioxidant factors in rat testis.

Parameter	Control	T-2	tBHQ+T-2	M
Testicles weight (g)	1.14 + 0.04 ^a^	0.96 + 0.01 ^b^	1.14 + 0.02 ^a^	1.11 + 0.04 ^b^
Sperm motility (%)	40.73 ± 2.11 ^a^	20.14 ± 0.42 ^b^	41.65 ± 1.84 ^a^	37.56 ± 1.47 ^a^
SOD (U/mg prot)	45.74 ± 0.42 ^a^	44.01 ± 1.71 ^a^	45.64 ± 0.59 ^a^	63.13 ± 1.53 ^b^
CAT (U/mg prot)	99.14 ± 1.23 ^a^	90.17 ± 0.43 ^b^	101.64 ± 0.45 ^a^	99.99 ± 0.80 ^a^
GSH-PX (U/mg)	36.88 ± 0.56 ^a^	32.18 ± 1.18 ^b^	35.31 ± 0.73 ^a^	35.70 ± 0.71 ^a^
TAC (nmol/g)	70.33 ± 0.35 ^a^	67.63 ± 0.69 ^b^	69.47 ± 0.71 ^a^	71.60 ± 0.64 ^a^
MDA (nmol/g prot)	235.57 ± 3.08 ^a^	295.8 ± 6.82 ^b^	158.17 ± 3.47 ^ab^	175.5 ± 2.16 ^ab^

tBHQ: tert-butylhydroquinone, T-2: T-2 toxin; The relative weight of testes = the total weight of testes (g)/body weight (g) × 100%, n = 10. Different letters represent significant differences *p* < 0.05.

## Data Availability

The remaining data are presented in the manuscript.

## Supplementary Materials

The following supporting information can be downloaded at: https://www.mdpi.com/article/10.3390/toxics12050335/s1, Table S1: Sequence of gene.
