# Supplementary material for: Tert-Butylhydroquinone Mitigates T-2-Toxin-Induced Testicular Dysfunction by Targeting Oxidative Stress, Inflammation, and Apoptosis in Rats
Source: Toxics. 2024 May 5;12(5):335. doi: 10.3390/toxics12050335 (PMC11125982; doi:10.3390/toxics12050335)
Supplement: Supplementary file 1 [file toxics-12-00335-s001.zip › toxics-2989540-supplementary.pdf]

Table S1. Sequence of gene.

Gene	Accession Number	Primer Sequence		Amplicon Size (bp)
GAPDH	NM-017008	FTCACCACCATGGAGAAGGC	RGCTAAGCAGTTGGTGGTGCA	169
PIK3	NM_001371300.2	FCCACGACGATTGCTCAA	RAGCCTGCACAGGAGTAA	150
caspase-3	NM_012922.2	FCCGGGTGCGGTAGAGTAAGC	RCTGGACTGCGGTATTGAGAC	104
TNF-α	NM_012675.3	FACTGAACTTCGGGGTGATCG	RGCTTGGTGGTTTGCTACGAC	153
IL-1β	NM_031512.2	FGACTTCACCATGGAACCCGT	RGGAGACTGCCCATTCTCGAC	104
IL-10	NM_012854.2	FTTGAACCACCCGGCATCTAC	RCCAAGGAGTTGCTCCCGTTA	91
Bax	U49729.1	FCGCGTGGTTGCCCTCTTCTACTTT	RCAAGCAGCCGCTCACGGAGGA	124
Bcl-2	NM_016993.1	FATCGCTCTGTGGATGACTGAGTAC	RAGAGACAGCCAGGAGAAATCAAAC	134
Nrf2	NM_031789.1	FCAGGTTGCCCACATTCCCAA	RATATCCAGGGCAAGCGACTCA	110
GnRH	NM_012767.2	FGCCGCTGTTGTTCTGTTGAC	RCTGGGGTTCTGCCATTTGA	153
LHβ	NM_012858.2	FCATAGTCTCCTTTCCTGTGGC	RCATTGGTTGAGTCCTGGGA	91
FSHβ	NM_001007597.2	FAGACCAAACACCCAGAAAG	RTCACTATCACACTTGCCACA	140
**GnrhR**	**NM_031038.3**	**FAGCCATCAACAACAGCATCC**	**RTCTTCTGGGTCCACCTCTGC**	**155**
**FshR**	**NM_199237.2**	**FCATAAACATCCACATCGTTGCC**	**RGTTCATCTAGCTGAGTTCCGTTG**	**131**
**LhR**	**NM_012978.2**	**FAACCTGCTATACATTGAACC**	**RGAGATTAGAGTCGTCCCATT**	**275**
Star	NM_031558.3	FGGGCATACTCAACAACCAG	RACCTCCAGTCGGAACACC	111
Cyp11a1	NM_017286.3	FCTTTGGTGCAGGTGGCTAG	RCGGAAGTGCGTGGTGTTT	115
3β-Hsd	NM_012584.2	FTGTGCCAGCCTTCATCTAC	RCTTCTCGGCCATCCTTTT	145
17β-Hsd	NM_054007.1	FGACCGCCGATGAGTTTGT	RTTTGGGTGGTGCTGCTGT	140
Cyp17a1	NM_012753.3	FTGGCTTTCCTGGTGCACAATC	RTGAAAGTTGGTGTTCGGCTGAAG	90
SOD	X05634.1	FCGAGCATGGGTTCCATGTC	RCTGGACCGCCATGTTTCTTAG	101
CAT	NM_012520.2	FACAACTCCCAGAAGCCTAAGAATG	RGCTTTTCCCTTGGCAGCTATG	76
GPx	NM_030826.4	FGGAGAATGGCAAGAATGAAGA	RCCGCAGGAAGGTAAAGAG	139
Tp53	NM_030989.3	FCTACTAAGGTCGTGAGACGCTGCC	RTCAGCATACAGGTTTCCTTCCACC	106
Caspase-9	NM_031632	FCTGAGCCAGATGCTGTCCCATA	RCCAAGGTCTCGATGTACCAGGAA	168

GAPDH: glyceraldehyde-3-phosphate dehydrogenase, PIK3: phosphatidylinositol 3-kinase , caspase-3:caspase-3,TNF: tumour necrosis factor,:IL: interleukin,

Bax: Bcl-2-associated X protein, Bcl-2: beta cell lymphoma-2, Nrf2: nuclear factor erythroid 2–related factor 2, GnRH:gonadotropin releasing hormone, LHβ:luteinizing hormone subunit beta,FSHβ: follicle stimulating hormone subunit beta, **GnrhR: gonadotropin releasing hormone receptor,** Star:steroidogenic acute regulatory protein,Cyp11a1:cytochrome P450, family 11, subfamily a, polypeptide 1, 3β-Hsd: hydroxy-delta-5-steroid dehydrogenase, 3 beta,17β-Hsd:hydroxysteroid (17-beta) , SOD: superoxide dismutase, CAT: catalase, GPx: glutathione peroxidase,   Tp53: tumour protein, Caspase-9:caspase 9.
